# 2-Amino-4-methyl­pyrimidinium dihydrogen phosphate

**DOI:** 10.1107/S160053681300648X

**Published:** 2013-03-13

**Authors:** Sajesh P. Thomas, Jyothi Sunkari

**Affiliations:** a Solid State and Structural Chemistry Unit, Indian Institute of Science, Bangalore 560 012, Karnataka, India; b Department of Chemistry, Kakatiya University, Warangal 506 009, India

## Abstract

A charge-assisted hydrogen-bonding network involving N—H⋯O and O—H⋯O hydrogen bonds stabilizes the crystal of the title salt, C_5_H_8_N_3_^+^·H_2_PO_4_^−^. The dihydrogen phosphate anions form one-dimensional chains along [100], *via* O—H⋯O hydrogen bonds. The 2-amino-4-methyl­pyrimidinium cations are linked to these chains by means of two different kinds of N—H⋯O hydrogen bonds. Neighbouring chains are linked *via* C—H⋯N and C—H⋯O hydrogen bonds forming two-dimensional slab-like networks lying parallel to (01-1).

## Related literature

Intriguing anion clusters formed by the supra­molecular assembly of dihydrogen phosphates have been investigated recently (see: Hossain *et al.*, 2012 ▶). Methyl­pyrimidine derivatives are known to be synthetic precursors to many bioactive pyrimidine derivatives (see: Xue *et al.*, 1993 ▶). Metal complexes of pyrimidines (see: Zhu *et al.*, 2008 ▶) and their proton transfer complexes with mineral acids are reported (see: Aakeroy *et al.*, 2003 ▶). The infinite O—H⋯O hydrogen-bond chain present in this material is a structural feature suggestive of possible proton conducting behaviour (see: Haile *et al.*, 2001 ▶).
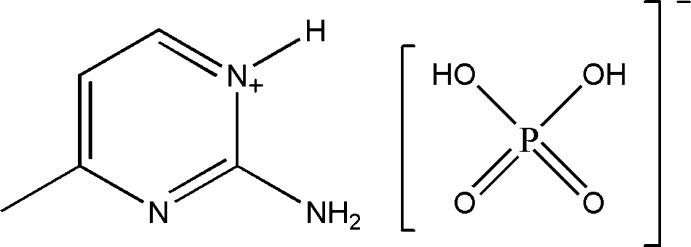


## Experimental

### 

#### Crystal data

C_5_H_8_N_3_^+^·H_2_PO_4_^−^
*M* *_r_* = 207.13
Triclinic, 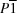

*a* = 6.1720 (2) Å
*b* = 7.5616 (3) Å
*c* = 9.9216 (4) Å
α = 100.562 (3)°
β = 99.821 (3)°
γ = 102.279 (4)°
*V* = 434.07 (3) Å^3^
*Z* = 2
Mo *K*α radiation
μ = 0.31 mm^−1^
*T* = 295 K
0.25 × 0.20 × 0.18 mm

#### Data collection

Oxford Xcalibur Eos (Nova) CCD detector diffractometer
Absorption correction: multi-scan (*CrysAlis RED*; Oxford Diffraction, 2006 ▶) *T*_min_ = 0.928, *T*_max_ = 0.947
9718 measured reflections
1717 independent reflections
1546 reflections with *I* > 2σ(*I*)
*R*_int_ = 0.027

#### Refinement

*R*[*F* ^2^ > 2σ(*F* ^2^)] = 0.031
*wR*(*F* ^2^) = 0.088
*S* = 1.08
1717 reflections
125 parameters
H atoms treated by a mixture of independent and constrained refinement
Δρ_max_ = 0.21 e Å^−3^
Δρ_min_ = −0.34 e Å^−3^

### 

Data collection: *CrysAlis CCD* (Oxford Diffraction, 2006 ▶); cell refinement: *CrysAlis PRO* (Oxford Diffraction, 2006 ▶); data reduction: *CrysAlis RED* (Oxford Diffraction, 2006 ▶); program(s) used to solve structure: *SHELXS97* (Sheldrick, 2008 ▶); program(s) used to refine structure: *SHELXL97* (Sheldrick, 2008 ▶) and *WinGX* (Farrugia, 2012 ▶); molecular graphics: *Mercury* (Macrae *et al.*, 2008 ▶); software used to prepare material for publication: *PLATON* (Spek, 2009 ▶).

## Supplementary Material

Crystal structure: contains datablock(s) global, I. DOI: 10.1107/S160053681300648X/ds2227sup1.cif

Structure factors: contains datablock(s) I. DOI: 10.1107/S160053681300648X/ds2227Isup2.hkl

Supplementary material file. DOI: 10.1107/S160053681300648X/ds2227Isup3.cml

Additional supplementary materials:  crystallographic information; 3D view; checkCIF report

## Figures and Tables

**Table 1:** Hydrogen-bond geometry (Å, °)

*D*—H⋯*A*	*D*—H	H⋯*A*	*D*⋯*A*	*D*—H⋯*A*
O1—H1⋯O4^i^	0.82	1.80	2.6100 (18)	168
N1—H1*N*⋯O2^ii^	0.86	2.14	3.000 (2)	177
O2—H2⋯O4^iii^	0.82	1.80	2.5843 (17)	161
N1—H2*N*⋯O3^iv^	0.86	2.01	2.845 (2)	163
N3—H3*N*⋯O3^ii^	0.90 (2)	1.73 (2)	2.6276 (19)	173 (2)
C4—H4⋯N2^v^	0.93	2.55	3.463 (2)	166
C5—H5*B*⋯O1^vi^	0.96	2.58	3.531 (3)	171

Symmetry codes: (i) 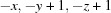; (ii) 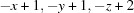; (iii) 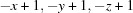; (iv) 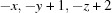; (v) 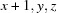; (vi) 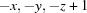.

**Fig. 1.**
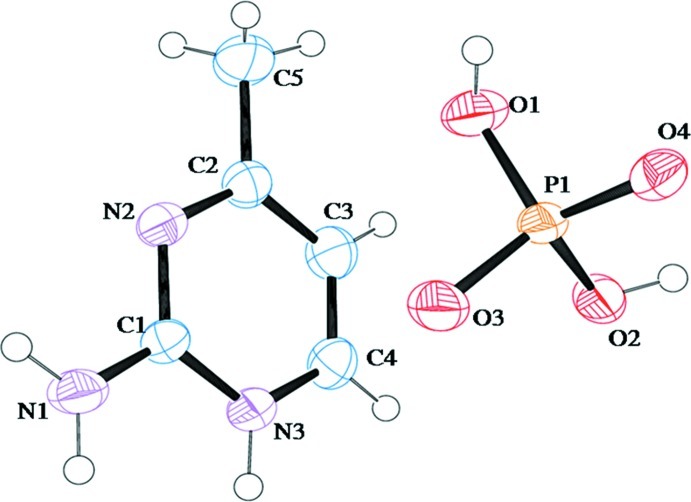
ORTEP view of the title compound with the atom numbering scheme. Displacement ellipsoids for non-H atoms are drawn at the 50% probability level.

**Fig. 2.**
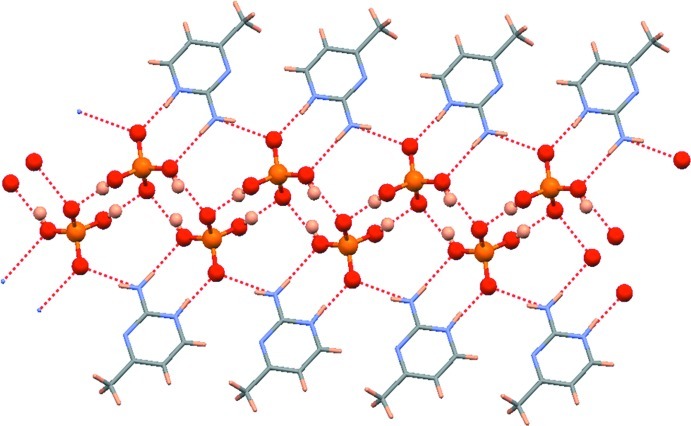
A view of supramolecular chain showing the hydrogen bonding between dihydrogen phosphate residues and the interlinked 2-amino-4-methylpyrimidine molecules.

## supplementary crystallographic information

### Comment

The title compound, a multicomponent crystal, AMHP, crystallizes in triclinic P-1, with a protonated 2-amino-4-methylpyrimidine molecule and a dihydrogenphosphate moiety in the asymmetric unit (Fig. 1). The dihydrogenphosphate residue forms a chain *via* O—H···O hydrogen bonds. 2-Amino-4-methylpyrimidinium cations are linked to these chains by means of two different kinds of N—H···O hydrogen bonds. The crystal packing is stabilized by N—H···O and O—H···O hydrogen bonds and the resulting supramolecular assembly is shown in Figure 2. The infinite hydrogen bond chains present in this structure are of special interest due to the anticipated proton conductivity of the material (see: Haile *et al.*2001).

### Experimental

The title compound was prepared by treating 2-amino 4-methyl pyramidine with phosphoric acid (H3PO4) in aqueous solution (Scheme 1) in 1:1 molar ratio. The crystals were harvested from the solution after 10 days and suitable crystal for single-crystal X-ray diffraction study were chosen using a polarizing microscope.

### Refinement

Data collection: *CrysAlis CCD* (Oxford Diffraction, 2006); cell refinement: *CrysAlis PRO* (Oxford Diffraction, 2006); data reduction: *CrysAlis RED* (Oxford Diffraction, 2006); program(*s*) used to solve structure: *SHELXL97* (Sheldrick, 2008); program(*s*) used to refine structure: *SHELXL97* (Sheldrick, 2008), *WinGX*(Farrugia, 2012); molecular graphics: Mercury 2.3 (Macrae *et al.* 2008); software used to prepare material for publication: *PLATON* (Spek, 2009).

### Crystal data

**Table:**

C_5_H_8_N_3_^+^·H_2_PO_4_^−^	*Z* = 2
*M**_r_* = 207.13	*F*(000) = 216
Triclinic, *P*1	Least Squares Treatment of 25 SET4 setting angles.
Hall symbol: -P 1	*D*_x_ = 1.585 Mg m^−^^3^
*a* = 6.1720 (2) Å	Mo *K*α radiation, λ = 0.71073 Å
*b* = 7.5616 (3) Å	Cell parameters from 326 reflections
*c* = 9.9216 (4) Å	θ = 2.8–26.0°
α = 100.562 (3)°	µ = 0.31 mm^−^^1^
β = 99.821 (3)°	*T* = 295 K
γ = 102.279 (4)°	Block, colourless
*V* = 434.07 (3) Å^3^	0.25 × 0.20 × 0.18 mm

### Data collection

**Table:**

Oxford Xcalibur Eos (Nova) CCD detector diffractometer	1717 independent reflections
Radiation source: Enhance (Mo) X-ray Source	1546 reflections with *I* > 2σ(*I*)
Graphite monochromator	*R*_int_ = 0.027
ω scans	θ_max_ = 26.0°, θ_min_ = 2.8°
Absorption correction: multi-scan (*CrysAlis RED*; Oxford Diffraction, 2006)	*h* = −7→7
*T*_min_ = 0.928, *T*_max_ = 0.947	*k* = −9→9
9718 measured reflections	*l* = −12→12

### Refinement

**Table:**

Refinement on *F*^2^	Primary atom site location: structure-invariant direct methods
Least-squares matrix: full	Secondary atom site location: difference Fourier map
*R*[*F*^2^ > 2σ(*F*^2^)] = 0.031	Hydrogen site location: inferred from neighbouring sites
*wR*(*F*^2^) = 0.088	H atoms treated by a mixture of independent and constrained refinement
*S* = 1.08	*w* = 1/[σ^2^(*F*_o_^2^) + (0.0449*P*)^2^ + 0.1805*P*] where *P* = (*F*_o_^2^ + 2*F*_c_^2^)/3
1717 reflections	(Δ/σ)_max_ < 0.001
125 parameters	Δρ_max_ = 0.21 e Å^−^^3^
0 restraints	Δρ_min_ = −0.34 e Å^−^^3^

### Special details

**Table:**

Geometry. Bond distances, angles *etc*. have been calculated using the rounded fractional coordinates. All su's are estimated from the variances of the (full) variance-covariance matrix. The cell e.s.d.'s are taken into account in the estimation of distances, angles and torsion angles
Refinement. Refinement on *F*^2^ for ALL reflections except those flagged by the user for potential systematic errors. Weighted *R*-factors *wR* and all goodnesses of fit *S* are based on *F*^2^, conventional *R*-factors *R* are based on *F*, with *F* set to zero for negative *F*^2^. The observed criterion of *F*^2^ > σ(*F*^2^) is used only for calculating -*R*-factor-obs *etc*. and is not relevant to the choice of reflections for refinement. *R*-factors based on *F*^2^ are statistically about twice as large as those based on *F*, and *R*-factors based on ALL data will be even larger.

### Fractional atomic coordinates and isotropic or equivalent isotropic displacement parameters (Å^2^)

**Table:**

	*x*	*y*	*z*	*U*_iso_*/*U*_eq_	
N1	0.1136 (3)	0.3869 (2)	1.11795 (16)	0.0405 (5)	
N2	−0.0464 (2)	0.1740 (2)	0.90738 (15)	0.0332 (4)	
N3	0.3480 (2)	0.2530 (2)	0.99937 (15)	0.0326 (4)	
C1	0.1379 (3)	0.2712 (2)	1.00803 (17)	0.0293 (5)	
C2	−0.0146 (3)	0.0613 (2)	0.79698 (18)	0.0341 (5)	
C3	0.2007 (3)	0.0371 (3)	0.7852 (2)	0.0407 (6)	
C4	0.3796 (3)	0.1362 (3)	0.88869 (19)	0.0386 (6)	
C5	−0.2202 (4)	−0.0381 (3)	0.6843 (2)	0.0507 (7)	
P1	0.28422 (7)	0.48498 (6)	0.64883 (4)	0.0283 (1)	
O1	0.0685 (2)	0.31898 (17)	0.59152 (14)	0.0418 (4)	
O2	0.4901 (2)	0.39299 (18)	0.64980 (12)	0.0365 (4)	
O3	0.2949 (2)	0.5709 (2)	0.79898 (13)	0.0439 (4)	
O4	0.29115 (19)	0.61556 (16)	0.55043 (13)	0.0336 (4)	
H1N	0.23060	0.44920	1.18240	0.0490*	
H2N	−0.01930	0.40020	1.12510	0.0490*	
H3	0.21960	−0.04470	0.70830	0.0490*	
H3N	0.464 (4)	0.319 (3)	1.071 (2)	0.048 (6)*	
H4	0.52500	0.12390	0.88380	0.0460*	
H5A	−0.31890	−0.12620	0.71950	0.0760*	
H5B	−0.17500	−0.10210	0.60520	0.0760*	
H5C	−0.29890	0.05020	0.65580	0.0760*	
H1	−0.03370	0.35420	0.54850	0.0630*	
H2	0.53240	0.39280	0.57580	0.0550*	

### Atomic displacement parameters (Å^2^)

**Table:**

	*U*^11^	*U*^22^	*U*^33^	*U*^12^	*U*^13^	*U*^23^
N1	0.0258 (7)	0.0530 (9)	0.0348 (8)	0.0121 (7)	0.0011 (6)	−0.0069 (7)
N2	0.0252 (7)	0.0384 (8)	0.0319 (7)	0.0049 (6)	0.0049 (6)	0.0030 (6)
N3	0.0238 (7)	0.0423 (8)	0.0309 (7)	0.0087 (6)	0.0047 (6)	0.0069 (6)
C1	0.0241 (8)	0.0338 (8)	0.0303 (8)	0.0081 (6)	0.0052 (6)	0.0081 (7)
C2	0.0327 (9)	0.0334 (8)	0.0331 (9)	0.0033 (7)	0.0079 (7)	0.0048 (7)
C3	0.0410 (10)	0.0435 (10)	0.0373 (10)	0.0132 (8)	0.0140 (8)	0.0003 (8)
C4	0.0315 (9)	0.0494 (10)	0.0403 (10)	0.0173 (8)	0.0134 (8)	0.0103 (8)
C5	0.0393 (11)	0.0546 (12)	0.0424 (11)	−0.0023 (9)	0.0047 (8)	−0.0077 (9)
P1	0.0201 (2)	0.0388 (3)	0.0242 (2)	0.0076 (2)	0.0030 (2)	0.0046 (2)
O1	0.0294 (7)	0.0421 (7)	0.0492 (8)	0.0029 (5)	−0.0041 (6)	0.0177 (6)
O2	0.0300 (6)	0.0563 (8)	0.0293 (6)	0.0201 (6)	0.0081 (5)	0.0126 (6)
O3	0.0298 (7)	0.0705 (9)	0.0280 (7)	0.0165 (6)	0.0057 (5)	−0.0015 (6)
O4	0.0238 (6)	0.0396 (6)	0.0379 (7)	0.0078 (5)	0.0066 (5)	0.0106 (5)

### Geometric parameters (Å, º)

**Table:**

P1—O3	1.4964 (13)	N1—H2N	0.8600
P1—O1	1.5623 (14)	N1—H1N	0.8600
P1—O2	1.5725 (14)	N3—H3N	0.90 (2)
P1—O4	1.5098 (13)	C2—C5	1.492 (3)
O1—H1	0.8200	C2—C3	1.401 (3)
O2—H2	0.8200	C3—C4	1.347 (3)
N1—C1	1.319 (2)	C3—H3	0.9300
N2—C2	1.329 (2)	C4—H4	0.9300
N2—C1	1.349 (2)	C5—H5C	0.9600
N3—C1	1.348 (2)	C5—H5A	0.9600
N3—C4	1.347 (2)	C5—H5B	0.9600
			
P1···H3N^i^	2.89 (2)	C2···C1^ix^	3.481 (2)
P1···H2N^ii^	3.1000	C2···O1	3.099 (2)
P1···H1^iii^	2.8900	C3···O1	3.257 (2)
P1···H1N^i^	3.0600	C4···O2	3.395 (2)
P1···H2^iv^	2.8800	C5···O1	3.277 (3)
O1···C3	3.257 (2)	C5···H4^viii^	2.9800
O1···C5	3.277 (3)	H1···P1^iii^	2.8900
O1···O4^iii^	2.6100 (18)	H1···O4^iii^	1.8000
O1···C2	3.099 (2)	H1···H1^iii^	2.5500
O2···N1^i^	3.000 (2)	H1N···H2^i^	2.5200
O2···C4	3.395 (2)	H1N···H3N	2.2400
O2···O4^iv^	2.5843 (17)	H1N···P1^i^	3.0600
O3···N1^ii^	2.845 (2)	H1N···O2^i^	2.1400
O3···N3^i^	2.6276 (19)	H2···O4^iv^	1.8000
O4···O1^iii^	2.6100 (18)	H2···H1N^i^	2.5200
O4···O2^iv^	2.5843 (17)	H2···H2^iv^	2.4500
O1···H5B^v^	2.5800	H2···P1^iv^	2.8800
O2···H1N^i^	2.1400	H2N···P1^ii^	3.1000
O3···H3N^i^	1.73 (2)	H2N···O3^ii^	2.0100
O3···H2N^ii^	2.0100	H3···O4^x^	2.9100
O4···H2^iv^	1.8000	H3···H5B	2.3900
O4···H1^iii^	1.8000	H3N···H1N	2.2400
O4···H3^vi^	2.9100	H3N···P1^i^	2.89 (2)
O4···H5A^vii^	2.8000	H3N···O3^i^	1.73 (2)
N1···O2^i^	3.000 (2)	H4···N2^xi^	2.5500
N1···O3^ii^	2.845 (2)	H4···C5^xi^	2.9800
N3···O3^i^	2.6276 (19)	H5A···O4^xii^	2.8000
N2···H4^viii^	2.5500	H5B···H3	2.3900
C1···C2^ix^	3.481 (2)	H5B···O1^v^	2.5800
			
O3—P1—O4	115.79 (8)	N2—C1—N3	121.54 (15)
O1—P1—O3	109.84 (8)	N2—C2—C3	122.14 (16)
O1—P1—O4	109.55 (7)	N2—C2—C5	116.73 (17)
O1—P1—O2	104.78 (7)	C3—C2—C5	121.13 (17)
O2—P1—O4	110.13 (7)	C2—C3—C4	117.84 (18)
O2—P1—O3	106.13 (7)	N3—C4—C3	120.03 (18)
P1—O1—H1	109.00	C2—C3—H3	121.00
P1—O2—H2	109.00	C4—C3—H3	121.00
C1—N2—C2	117.90 (15)	C3—C4—H4	120.00
C1—N3—C4	120.52 (15)	N3—C4—H4	120.00
H1N—N1—H2N	120.00	C2—C5—H5B	109.00
C1—N1—H1N	120.00	C2—C5—H5C	109.00
C1—N1—H2N	120.00	C2—C5—H5A	109.00
C1—N3—H3N	117.5 (15)	H5A—C5—H5C	109.00
C4—N3—H3N	121.9 (15)	H5B—C5—H5C	109.00
N1—C1—N3	118.80 (16)	H5A—C5—H5B	110.00
N1—C1—N2	119.66 (17)		
			
C2—N2—C1—N1	178.81 (15)	C4—N3—C1—N2	0.1 (2)
C2—N2—C1—N3	−1.3 (2)	C1—N3—C4—C3	0.3 (3)
C1—N2—C2—C3	2.1 (2)	N2—C2—C3—C4	−1.7 (3)
C1—N2—C2—C5	−177.38 (16)	C5—C2—C3—C4	177.75 (19)
C4—N3—C1—N1	−179.98 (18)	C2—C3—C4—N3	0.4 (3)

Symmetry codes: (i) −*x*+1, −*y*+1, −*z*+2; (ii) −*x*, −*y*+1, −*z*+2; (iii) −*x*, −*y*+1, −*z*+1; (iv) −*x*+1, −*y*+1, −*z*+1; (v) −*x*, −*y*, −*z*+1; (vi) *x*, *y*+1, *z*; (vii) *x*+1, *y*+1, *z*; (viii) *x*−1, *y*, *z*; (ix) −*x*, −*y*, −*z*+2; (x) *x*, *y*−1, *z*; (xi) *x*+1, *y*, *z*; (xii) *x*−1, *y*−1, *z*.

### Hydrogen-bond geometry (Å, º)

**Table:**

*D*—H···*A*	*D*—H	H···*A*	*D*···*A*	*D*—H···*A*
O1—H1···O4^iii^	0.82	1.80	2.6100 (18)	168
N1—H1*N*···O2^i^	0.86	2.14	3.000 (2)	177
O2—H2···O4^iv^	0.82	1.80	2.5843 (17)	161
N1—H2*N*···O3^ii^	0.86	2.01	2.845 (2)	163
N3—H3*N*···O3^i^	0.90 (2)	1.73 (2)	2.6276 (19)	173 (2)
C4—H4···N2^xi^	0.93	2.55	3.463 (2)	166
C5—H5*B*···O1^v^	0.96	2.58	3.531 (3)	171

Symmetry codes: (i) −*x*+1, −*y*+1, −*z*+2; (ii) −*x*, −*y*+1, −*z*+2; (iii) −*x*, −*y*+1, −*z*+1; (iv) −*x*+1, −*y*+1, −*z*+1; (v) −*x*, −*y*, −*z*+1; (xi) *x*+1, *y*, *z*.
